# Machine learning applications in predicting pharmacological treatment outcomes and responses in esophageal cancer

**DOI:** 10.3389/fphar.2026.1786601

**Published:** 2026-06-17

**Authors:** Zhijing Yan, Yaoting Zhou, Shuyi Jia, Carolina Oi Lam Ung, Menghuan Song, Yunfeng Lai, Hao Hu, Yue Yang

**Affiliations:** 1 School of Pharmaceutical Sciences, Tsinghua University, Beijing, China; 2 State Key Laboratory of Mechanism and Quality of Chinese Medicine, University of Macau, Macau, Macao SAR, China; 3 Center of Excellence in Translational and Regulatory Sciences, School of Pharmaceutical Sciences, Tsinghua University, Beijing, China; 4 Centre for Pharmaceutical Regulatory Sciences, University of Macau, Macau, Macao SAR, China; 5 Department of Public Health and Medicinal Administration, Faculty of Health Sciences, University of Macau, Macau, Macao SAR, China; 6 School of Public Health and Management, Guangzhou University of Chinese Medicine, Guangzhou, China

**Keywords:** esophageal cancer, machine learning, pharmacotherapy, systematic review, treatment outcome

## Abstract

**Introduction:**

This study evaluated machine learning (ML) models predicting esophageal cancer (EC) treatment outcomes, focusing on data modalities, feature engineering, model frameworks, and validation.

**Methods:**

Following PRISMA guidelines (PROSPERO: CRD42024619947), six databases (2015–2024) were systematically searched. Two reviewers independently extracted data on model methodologies and performance. Study quality was assessed using a modified TRIPOD (Transparent Reporting of a multivariable prediction model for Individual Prognosis Or Diagnosis) + AI checklist.

**Results:**

Among 30 studies (14,342 patients), classical ML models were the most frequently employed approach (n = 43), followed by ensemble methods (n = 34), with deep learning being the least utilized (n = 11); however, the best-performing models across all studies demonstrated mean AUC values of 0.847 for deep learning, 0.835 for ensemble models, and 0.816 for classical approaches. Imaging and clinical data constituted the predominant both unimodal and multimodal modeling inputs, with supervised learning representing the dominant paradigm. Multimodal models achieved a significantly higher AUC (0.84 vs. 0.78) than single-modal models. Model validation primarily relied on k-fold cross-validation and external cohort approaches. Quality assessment showed moderate reporting completeness (64.79% median fulfillment).

**Discussion:**

While ML (particularly deep learning and multimodal approaches) demonstrated potential for EC treatment prediction, key limitations persisted, such as opaque computational methods, poorly justified predictor selection, and unaddressed population heterogeneity/class imbalance. Addressing these challenges would be critical to enhancing the reliability and clinical applicability of ML models in future research.

## Graphical Abstract

**Figure:**
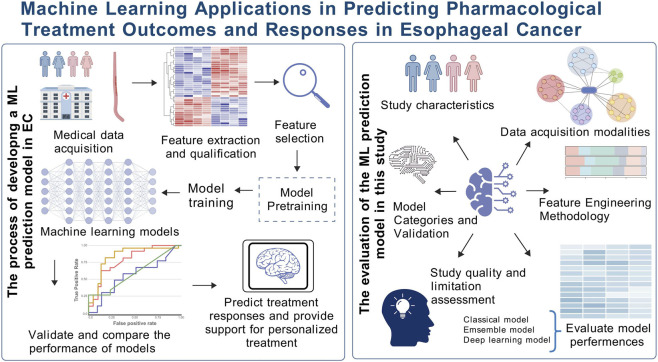
This study develops a machine learning (ML) model to predict esophageal cancer (EC) treatment response, systematically comparing methodologies and algorithmic performance. Key steps include multimodal data extraction (imaging, clinical, lab), feature selection, and model training, with pretrained models as alternatives for limited datasets. The analysis evaluated: (1) study baselines, (2) single- vs. multimodal inputs, (3) feature engineering, (4) ML model categories (classical/ensemble/deep learning), and (5) validation strategies. Standardized checklists assessed study quality and limitations.

## 1 Introduction

In 2022, a total of 511,054 new cases and 445,391 deaths were estimated globally, indicating that esophageal cancer (EC) was the seventh leading cause of cancer-related mortalities worldwide. A detailed analysis of these statistics showed that 74.9% of new EC occurred in Asia, with 327,700 incident cases in East Asia versus 59,500 in Europe in 2021 (Ferlay et al., 2025; Ajani et al., 2023; Jin et al., 2025). Pharmacological therapies are widely prescribed and play an essential role in the treatment of esophageal cancer. Neoadjuvant radiotherapy (NCRT) followed by esophagectomy has become the mainstay of treatment for patients with locally advanced resectable esophageal cancer (Yang et al., 2018; Shapiro et al., 2015). For patients with advanced EC who are unable to undergo resective surgery, receiving chemotherapy and/or targeted therapy, despite an increase in adverse events, still delivers benefits over optimal supportive care alone in terms of prolonging the patient’s overall survival and improving quality of life (Janmaat et al., 2017). Immune checkpoint inhibitors have demonstrated encouraging efficacy and an acceptable safety profile in patients with gastro-esophageal cancer, particularly in those with biomarker-positive diagnoses, as indicated by the most recent relevant clinical treatment guidelines from the American National Comprehensive Cancer Network (NCCN) and the European Society for Medical Oncology (ESMO) (Ajani et al., 2023; Muro et al., 2019).

The rapid advancements in the field of artificial intelligence (AI) have prompted a considerable increase in the utilization of machine learning (ML) for a wide scope of detection, diagnostic, supervisory and curative procedures in contemporary healthcare. Machine learning models can be trained on processed clinical data, followed by testing and parameters adjustment, and validated for performance, ultimately contributing to the generation of accountable results for clinical references (de Lange et al., 2018). For example, the newly developed Deep lEarning from histoPathoLOgy and methYlation (DEPLOY) enabled precise diagnosis of diverse central nervous system tumor types (Hoang et al., 2024a). ENLIGHT-DeepPT was employed to predict transcriptomic profiles and therapeutic responses in patients from The Cancer Genome Atlas cohort using histopathological images (Hoang et al., 2024b). Compared to previous gene set enrichment-based approaches, predicTCR demonstrated superior capability in identifying tumor-reactive T cell receptors within tumor-infiltrating lymphocytes (TILs) across various cancer types, thereby facilitating future personalized T-cell therapies (Tan et al., 2025). These technologies now drive transformative progress in oncology, changing early diagnosis, molecular subtyping, and tailored interventions. Their transition from research to clinical practice is accelerating, with federated learning and multimodal large-scale models poised to further enhance diagnostic accessibility and precision.

Published studies have demonstrated the efficacy of ML in precision health. Its current application in EC primarily focuses on diagnostic medicine, particularly through imaging modalities, alongside reports on the safety and efficacy of robotic-assisted surgery (Ma et al., 2022; Zhou et al., 2022; Visaggi et al., 2022; Islam et al., 2022). However, the number of reviews systematically describing and summarizing the role of ML in predicting treatment outcome and response to drug therapy for EC remains limited.

Therefore, the primary objective of this study was to delineate the application of machine learning in predicting therapeutic outcomes and responses in EC, to summarize the current landscape of ML applications in forecasting drug treatment efficacy for EC, offering a comprehensive methodological framework encompassing data categories, feature selection strategies for model training, and model modalities. Furthermore, it sought to critically evaluate validation methodologies and assess the performance metrics of diverse model architectures. This study would benefit developers and potential end-users (e.g., clinicians and healthcare providers) interested in leveraging ML techniques for predicting tumor treatment outcomes.

## 2 Methods

### 2.1 Search strategy and eligibility criteria

This systematic review followed the latest version of the preferred reporting items for systematic reviews and meta-analyses (PRISMA) guideline for identifying potentially related articles (Moher et al., 2015). The protocol for this systematic review was prospectively registered at PROSPERO (CRD42024619947).

Six electronic databases, PubMed, Web of Science, IEEE Xplore, ACM Digital Library, Cochrane Library (Trials) and Scopus, were searched. Studies published in English from 1 January 2015 to 29 October 2024 were included. Subject headings and Medical Subject Headings (MeSH) terms were used in title and abstract search (Supplementary Table A1).

This systematic review employed the population, intervention, comparison, outcome, and study type (PICOS) framework to define eligibility criteria (Supplementary Table A2) (Schardt et al., 2007). For population, any adult patients undergoing pharmacological treatment (including chemotherapies, immunotherapies, neoadjuvant therapies and combination therapies with any drug treatments, such as radiochemotherapy) for EC, including esophageal adenocarcinoma (EAC) and esophageal squamous cell carcinoma (ESCC), were included. Both pre- and post- medication surgery, with and without surgery would be included in this study. Animal experiments and virtual data from computer simulations were excluded. The intervention was ML models applied for prediction of pharmacological treatment outcomes and responses in EC patients. The selection prioritized rigorous methodology and clinical relevance, with explicit exclusion of non-quantitative analyses, preclinical research, and non-peer-reviewed materials to ensure clinical translational validity and reproducibility in EC therapeutic decision-making contexts.

To clarify the technological landscape, we categorized the predictive models into three groups: classical machine learning, deep learning (DL), and ensemble models. Classical ML models (e.g., Support Vector Machine, Logistic Regression) primarily rely on traditional feature engineering, where clinical or imaging descriptors are manually extracted and selected. In contrast, DL models (e.g., Convolutional Neural Network [CNN], Artificial Neural Network [ANN]) utilize multi-layered architectures to automate hierarchical feature representation directly from raw data. Furthermore, we identified ensemble models (e.g., Random Forest, XGBoost, and stacking techniques) as a distinct category that combines multiple learners to improve predictive stability and accuracy (Ezugwu et al., 2025).

### 2.2 Study selection and data extraction

Two reviewers independently assessed the eligibility of the retrieved papers. A standardized dual-extraction was used to collect data from included studies, categorized into six domains: (1) basic study information, (2) data collection/preprocessing, (3) patient characteristics, (4) treatment protocols/outcomes, (5) ML methods (algorithms/validation), and (6) performance metrics, including accuracy, sensitivity (recall), specificity, area under the curve (AUC), positive predictive value (PPV; precision), negative predictive value (NPV), F1-score, and C-index values. The analysis compared unimodal and multimodal modality strategies, with emphasis on validation rigor (internal/external) for clinical generalizability. Any disagreements between the reviewers were resolved through discussion and consensus with a third reviewer.

### 2.3 Quality assessment

Two reviewers independently assessed the quality and risk of bias of the included reviews using a modified checklist based on Transparent Reporting of a multivariable prediction model for Individual Prognosis Or Diagnosis (TRIPOD) + AI (Supplementary Table A3) (Collins et al., 2024). Depending on weather each item was met or not reported, the reviewer would mark as “Yes”, “No” or “NR”. If a checklist item was “Yes”, it would be counted as one point. There was a total of 56 evaluation checklist items, totaling 56 points. Checklist items and scoring were excluded for questions that were not applicable in the study.

Studies were rated on the basis of methodological quality and evaluated for high (fulfillment higher than 66.7%), medium (fulfillment 33.4%–66.6%) or low (fulfillment below 33.3%) rigor according to a standardized assessment checklist modified from TRIPOD + AI (Collins et al., 2024; Alapati et al., 2025). In addition, Cohen’s Kappa coefficient (κ) were categorized according to the checklist questions in order to ascertain the inter-reviewer reliability as no agreement (≤0), slight agreement (0.01-0.20), fair agreement (0.21-0.40), moderate agreement (0.41-0.60), substantial agreement (0.61-0.80), near perfect agreement (0.81-0.99) or perfect agreement (1.00) (Tang et al., 2015).

### 2.4 Strategy for data analysis

We performed descriptive statistical analyses, including calculating the median and interquartile range (IQR). The primary outcome was AUC of the machine learning algorithm in predicting EC treatment outcomes and pathological response. Secondary outcomes included the algorithm’s accuracy, sensitivity, specificity, PPV, NPV, and C-index.

All statistical analyses were performed using R statistical software (version 4.3.3), while network weighting and visualization for modals data collection modalities were implemented in Gephi (version 0.10.1). Model performance metrics were computationally processed and graphically rendered using MATLAB R2024a.

## 3 Results

### 3.1 Literature searching results

**FIGURE 1:**
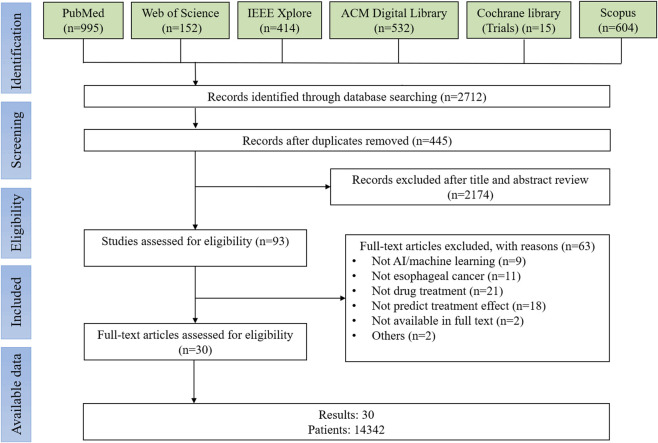
Process of literature screening.

A total of 2,712 records were identified in all databases searched. The titles and abstracts of 2,267 studies were screened following the removal of 445 duplicate articles. 2,174 articles found to be non-compliant were excluded, and the remaining 93 studies were assessed in full text. Sixty-three research were excluded after full text reading (Figure 1). Finally, a ground total of 30 researches employing ML algorithms to predict the pharmacological therapy outcome or response for EC patients were included in this review.

### 3.2 Characteristics of literature

**FIGURE 2:**
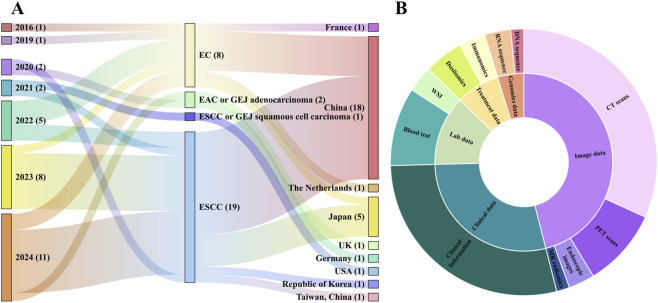
Comprehensive analysis of included studies. **(A)** Publication distribution of year, diseases and countries or regions; **(B)** Data category of the studies.

Over the last 10 years, there had been a consistent and significant growth in the volume of research publications focusing on machine learning-based predictive models for therapeutic outcomes in esophageal cancer (Figure 2). A total of 14,342 patients were included in these studies, most of whom were from East Asia (Chinese mainland: n = 18, Japan: n = 5) and some from Europe (n = 4) and America (n = 1). Except for two studies from United Kingdom (Rahman et al., 2020) and German (Jung et al., 2024) focused on adenocarcinoma, most studies included patients with ESCC or all types of EC, consistent with the global distribution of adenocarcinoma and squamous cell carcinoma in the literature (Sasagawa et al., 2022). The demographic composition of the patient population is as follows: 11,367 patients identified as male (79.26%), 2,647 as female (18.46%), and 178 patients were not referred to the gender category (1.24%). The mean age was 62.36 in published population information. Most of the studies were retrospective, with one study was a retrospective design from prospective trials. There were eighteen and ten studies conducted in single-center and two-center respectively, only two studies use multi-center data and two relied on database data. Approximately half of the studies (n = 14) had sample sizes ranging from 101 to 200. Eight studies reported sample sizes below 100, while five studies had sample sizes between 200 and 500. Huang et al. (2022) used data from both SEER database (n = 8,569) and a Chinese hospital database, encompassing a total of 9,096 patients.

### 3.3 Data acquisition modalities

Patient characteristics and clinical data strongly correlated with treatment outcomes formed the foundational basis for machine learning in precision medicine, encompassing clinical information (age, gender, medical history), imaging data (CT/PET scans), and laboratory results (blood biomarkers, whole slide images). ML models integrate these multidimensional datasets to uncover latent pathological patterns, supported by multi-omics data (genomics, doseomics, immunomics) enhancing predictive potential. Among EC treatment prediction models, image (n = 23) and clinical data (n = 18) were most prevalent, followed by lab (n = 8), treatment (n = 7), and genomics data (n = 2), with CT scans (n = 20) and clinical information (n = 18) as frequent inputs, while PET scans (n = 6), blood tests (n = 6), and omics data were less utilized (Figure 2). Multimodal models were computational or analytical frameworks that integrated and processed data from multiple sources or modalities. In total, 17 studies trained unimodal models and 21 articles trained multimodal models, some trained both unimodal and multimodal models (n = 8). The integration of these multimodal datasets enables ML models to decode non-linear interactions (Supplementary Tables A4, 5).

Comparative analyses of single-modality versus multi-modality training models revealed systematic performance disparities under identical algorithms. Among eight studies, one lacked single-modality AUC data; the remaining seven demonstrated a mean AUC of 0.78 (range: 0.725–0.817) for optimal single-modality models versus 0.84 (range: 0.796–0.891) for multi-modal counterparts, with unanimous consensus on the superiority of multimodal approaches.

### 3.4 Predictive modeling framework

#### 3.4.1 Model categories and paradigms

The most common algorithms employed in EC treatment prediction were SVM (n = 16) and LR (n = 12) of the classical ML models, as well as RF (n = 14) and XGBoost (n = 12) of the ensemble models. Although DL models were used less frequently, they exhibited a reliable ratio of best performance to the number of using times (7/11, 63.64%), significantly elevated in comparison to ensemble models (15/34, 44.12%) and classical models (9/44, 20.45%) (Table 1).

**TABLE 1:** The times for utilization and best performance of different categories of models.

​	Appearance of uses	Appearance of best performances	Total uses	Total best performances	Percentage of best performances/Uses
	Code	Code			
Classical ML models			44	9	20.45%
SVM	(Jin et al., 2019), (Hu et al., 2020) (Hu et al., 2021), (Yoon et al., 2021), (Beukinga et al., 2022), (Yue et al., 2022), (Li et al., 2023a), (Li et al., 2023b), (Oda et al., 2023), (Kasai et al., 2024), (Kawahara et al., 2024), (Li et al., 2024b), (Lin et al., 2024), (Liu et al., 2024a), (Qi et al., 2024), (Zhang et al., 2024)	(Hu et al., 2020), (Hu et al., 2021), (Beukinga et al., 2022), (Li et al., 2023b), (Qi et al., 2024)	16	5	31.25%
LR	(Rishi et al., 2021), (Yoon et al., 2021), (Beukinga et al., 2022), (Yue et al., 2022), (Li et al., 2023a), (Oda et al., 2023), (Wang et al., 2023), (Kasai et al., 2024), (Lin et al., 2024), (Liu et al., 2024a), (Qi et al., 2024), (Zhang et al., 2024)	(Rishi et al., 2021), (Oda et al., 2023), (Wang et al., 2023), (Zhang et al., 2024)	12	4	33.33%
KNN	(Yoon et al., 2021), (Beukinga et al., 2022), (Oda et al., 2023), (Kawahara et al., 2024), (Lin et al., 2024), (Liu et al., 2024a)	​	6	0	-
DT	(Sasagawa et al., 2022), (Li et al., 2023a), (Oda et al., 2023), (Kawahara et al., 2024), (Liu et al., 2024a)	​	5	0	-
NB	(Yoon et al., 2021), (Beukinga et al., 2022), (Kasai et al., 2024), (Li et al., 2024b)	​	4	0	-
ELR	Rahman et al. (2020)	​	1	0	-
DL Models			11	7	63.64%
D-AAE	Yue et al. (2022)	​	1	0	-
HD-Net	Yue et al. (2022)	​	1	0	-
MLDRL	Yue et al. (2022)	Yue et al. (2022)	1	1	100.00%
DeepSurv	Huang et al. (2022)	Huang et al. (2022)	1	1	100.00%
NN	(Beukinga et al., 2022), (Kawahara et al., 2022), (Yap et al., 2023), (Jung et al., 2024), (Kasai et al., 2024), (Kawahara et al., 2024), (Li et al., 2024a)	(Kawahara et al., 2022), (Yap et al., 2023), (Jung et al., 2024), (Kawahara et al., 2024), (Li et al., 2024a)	7	5	71.43%
-CNN	(Kawahara et al., 2022), (Yap et al., 2023), (Jung et al., 2024)	(Kawahara et al., 2022), (Yap et al., 2023), (Jung et al., 2024)	3	3	100.00%
-ANN	Kasai et al. (2024)	​	1	0	-
-RNN	Li et al. (2024a)	Li et al. (2024a)	1	1	100.00%
Ensemble models			34	15	44.12%
RF	(Paul et al., 2017), (Rahman et al., 2020), (Yoon et al., 2021), (Beukinga et al., 2022), (Sasagawa et al., 2022), (Yue et al., 2022), (Cheng et al., 2023), (Li et al., 2023a), (Oda et al., 2023), (Kasai et al., 2024), (Li et al., 2024b), (Lin et al., 2024), (Liu et al., 2024a), (Qi et al., 2024)	(Paul et al., 2017), (Sasagawa et al., 2022), (Cheng et al., 2023), (Li et al., 2023a), (Kasai et al., 2024), (Li et al., 2024b), (Liu et al., 2024a)	14	7	50.00%
XGBoost	(Jin et al., 2019), (Hu et al., 2020), (Rahman et al., 2020), (Yue et al., 2022), (Li et al., 2023a), (Oda et al., 2023), (Li et al., 2024b), (Lin et al., 2024), (Liu et al., 2024a), (Qi et al., 2024), (Wang et al., 2024), (Zhang et al., 2024)	(Jin et al., 2019), (Li et al., 2024b), (Lin et al., 2024), (Wang et al., 2024)	12	4	33.33%
LightGBM	Yoon et al. (2021)	​	1	0	-
EFM	Yue et al. (2022)	​	1	0	-
LFM	Yue et al. (2022)	​	1	0	-
CTDEPN	Huang et al. (2023)	Huang et al. (2023)	1	1	100.00%
Random Subspace Ensemble Model	Kawahara et al. (2024)	​	1	0	-
Stacking Ensemble Model	Su et al. (2024)	Su et al. (2024)	1	1	100.00%
Ensemble Combinations	(Rahman et al., 2020), (Yoon et al., 2021)	(Rahman et al., 2020), (Yoon et al., 2021)	2	2	100.00%
-ELR + RF + XGBoost	Rahman et al. (2020)	Rahman et al. (2020)	1	1	100.00%
-LR + SVM	Yoon et al. (2021)	Yoon et al. (2021)	1	1	100.00%

Abbreviations: SVM, support vector machine; LR, logistic regression; KNN, K-Nearest Neighbors; DT, decision tree; NB, naive bayes; ELR, elastic network regression; D-AAE, Disentangled-Multimodal adversarial autoencoder; HD-Net, HyperDense-Network; MLDRL, Multi-loss Disentangled Representation Learning; NN, neural network; CNN, convolutional neural network; ANN, artificial neural network; RNN, recurrent neural network; RF, random forest; XGBoost, Extreme Gradient Boosting; LightGBM, light gradient boosting; EFM, early fusion method; LFM, late fusion method; CTDEPN, combined treatment decision for efficacy and prognosis nomogram.

“Appearance of uses” denoted the frequency of model applications across studies, while “Appearance of best performances” counted instances where a model demonstrated top performance, regardless of whether the study employed single-model evaluation or comparative multi-model assessments.

Kawahara et al. (20254) used NN model. Among the study of Li Z. et al. (2024), RF and XGBoost demonstrated superior performance.

In the included studies, supervised learning dominated (n = 28/30, 90.3%), whereas weakly supervised (n = 1) and unsupervised learning (n = 2) were significantly less frequent, collectively accounting for 10%. This distribution reflects the medical diagnosis and prediction field’s stringent demands for input data reliability.

#### 3.4.2 Feature engineering methodology

This study systematically quantified feature extraction and selection in Supplementary Table A6 (median counts used for studies involving multiple experiments or modals). Initial extraction showed heterogeneity: ≤100 features (n = 4/30, 13.3%), >2000 (n = 5/30, 16.7%), with 1001-2000 most prevalent (n = 6/30, 20%), reflecting preference for large feature pools despite 7 studies not reporting totals (NR). Selection revealed strong reduction: ≤10 features (n = 12/30, 40%), ≤20 (n = 8/30, 26.7%), indicating interpretability focus. Only 2 studies retained 41-60 features (potentially ensemble-related), while one DL model used 96 (exploiting high-dimensional compatibility). Absence of 21-40 or 61-80 features suggests traditional methods avoid intermediate dimensions. Six studies omitted post-selection counts (NR), and one used image patches, indicating varied engineering approaches.

Manual tumor feature extraction was historically constrained by inter-observer variability and time-intensive workflows, but emerging machine learning-based auto-segmentation tools provided clinically viable alternatives. PyRadiomics (http://github.com/Radiomics/pyradiomics#readme), employed in 11 studies, is an open-source Python library for quantitative radiomic feature extraction (van Griethuysen et al., 2017). Zhang et al. used AccuContour (Manteia Technologies, http://www.manteiatech.com/index_en.html), a U-Net-based DCNN platform enabling automated segmentation of >100 clinical structures with standardized preprocessing (Zhang et al., 2024; Johnson et al., 2024).

#### 3.4.3 Model perfermences evaluation

**FIGURE 3:**
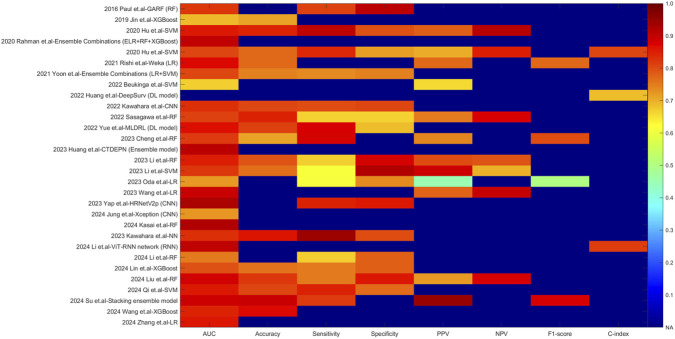
The heatmap of prediction ML models in EC treatment. Model selection criteria: (1) Choose the highest-performing prediction model from multiple candidates within a study; (2) Prioritize validation cohorts with both the largest sample size and most stable performance metrics. Model evaluation prioritization hierarchy: Testing > Validation > Training; External validation > Internal validation. In some studies, Recall (i.e., Sensitivity) and Precision (i.e., PPV) were used interchangeably. Rishi et al. (2021), Weka + LR used F-measure (which is in this figure regarded as F1-score). Weka was an integrated machine learning platform for data mining tasks. Among the study of Li et al. (2024), RF, Random Forest (RF) and XGBoost demonstrated superior performance, with RF selected as the optimal model based on AUC analysis.

This systematic analysis of 30 peer-reviewed studies (2016-2024) revealed that ensemble models and deep learning architectures were increasingly dominating the EC treatment prediction (Table 1), though classical ML models like Random Forest maintain competitive efficacy. The evaluation metrics exhibited significant variability across studies, with 53.3% (n = 16) reporting complete diagnostic parameters (AUC, sensitivity, specificity), a finding that underscored the need for standardized reporting per TRIPOD + AI guidelines. The performance of the optimal prediction models from each study was comprehensively summarized and visualized in Figure 3.

Model selection criteria: (1) Choose the highest-performing prediction model from multiple candidates within a study; (2) Prioritize validation cohorts with both the largest sample size and most stable performance metrics. Model evaluation prioritization hierarchy: Testing > Validation > Training; External validation > Internal validation. In some studies, Recall (i.e., Sensitivity) and Precision (i.e., PPV) were used interchangeably.

Among the optimal predictive models, ensemble models (14 studies, median AUC = 0.832, mean AUC = 0.835) demonstrated superior discriminative performance in EC prediction. Notably, Rahman et al. (2020) achieved AUC of 0.902 through strategic integration of elastic net regression (ELR) with RF and XGBoost algorithms. Paul et al. (2017) proposed genetic algorithm based on RF (GARF), a feature selection strategy with a fitness function combining RF misclassification rate, achieving 0.823 AUC (specificity = 0.91). The stacking ensemble framework (Su et al., 2024), incorporating five base learners and a meta-learner, attained 0.891 accuracy for 3-year survival prediction under limited sample conditions. Huang et al. (2023), developed Combined Treatment Decision for Efficacy and Prognosis Nomogram (CTDEPN), integrated radiomic and clinical parameters to dynamically score CCRT/RT options for elderly ESCC patients (≥65 years), enabling personalized treatment selection and achieving an AUC of 0.91.

Deep learning models (7 studies, median AUC = 0.848, mean AUC = 0.847) exhibited exceptional performance in complex pattern recognition tasks. Yap et al. (2023) developed a sophisticated computer-based methodology termed DCRNet, reached the highest AUC of 0.928 among all ML models evaluated in the study. This framework not only scrutinized medical images but also meticulously accounts for the spatial dispersion of therapeutic radiation doses within RT treatment plans, thereby yielding prognostications of markedly superior precision. Yue et al. (2022) proposed a multi-loss disentangled representation learning (MLDRL) method that enhances pCR prediction after NCRT by fusing longitudinal multi-stage data. The key innovations of the proposed method comprise: (1) feature disentanglement into inherent/variational components; (2) multi-loss optimization integrating reconstruction, separation, and classification; (3) adaptive gradient normalization. The model achieved 0.866 AUC and 0.875 sensitivity, surpassing single-stage and fusion benchmarks in both pCR prediction and unlabeled multicenter prognostic analysis. The study published the model construction formular and code (https://github.com/yuehailin/MLDRL.git). Huang et al. (2022) applied the DeepSurv DL survival prediction model for EC using 9,069 multicenter cases (SEER/CHINA), establishing an example of DL-based prognostic assessment and personalized treatment recommendations system for EC. Li B. et al. (2024) developed a ViT-RNN model predicting ESCC immunotherapy response from H&E slides, achieving 0.904 AUC and 0.814 C-index when combining pathomics signatures with PD-L1 expression for 6-month prognosis.

Classical ML models (n = 9, median AUC = 0.852, mean AUC = 0.816) were the most frequently employed approach across studies yet demonstrated fewer instances of superior performance compared to other model categories (Table 1). Rishi et al. (2021) utilized a Weka-based (machine learning algorithms for data mining tasks) dual-phase framework combining feature selection with logistic regression validation, balancing predictive accuracy and interpretability.

#### 3.4.4 Model validation profiling

As shown in Table 2, this review found k-fold cross-validation predominated (5-fold: n = 12; 10-fold: n = 5), with external validation widely used (external validation: n = 10; testing: n = 4). Other methods included hold-out (n = 8), stratified sampling, LOOCV and bootstrapping (each 1-2 studies), demonstrating methodological diversity in addressing distinct clinical prediction scenarios. Rahman et al. (2020) employed repeated 10-fold cross-validation across thousands of parameter sets, revealing performance disparities between training and validation phases, particularly for ensemble methods where single-run validation obscures overfitting risks. They augmented this with cross-institutional validation, implementing a two-phase nested internal-external framework that combines internal and external datasets for dual verification, quantitatively evaluating inter-institutional heterogeneity effects on model performance and demonstrating enhanced clinical relevance through realistic deployment simulation. Yoon et al. (2021) implemented two critical enhancements to the bootstrapping bias-corrected cross-validation (BBC-CV) methodology: firstly, they replaced the conventional bootstrap sampling with scikit-learn’s stratified train_test_split function to maintain proportional representation of clinically significant subgroups across training and test sets, thereby overcoming the small-sample representation bias inherent in standard BBC-CV’s resampling approach; secondly, they incorporated RandomOverSampler to dynamically adjust class distribution during each iteration, effectively addressing the prevalent class imbalance issue in clinical dataset.

**TABLE 2:** Characteristic of literature.

Basic information				Data collection	Patients	ML model						Availability	
Code	References	Study design	Study goal	Modality approach		Feature extract methods/ Platform	Feature selection methods	Type of validation	Best model	External validation	External testing	Data	Algorithm code
1	2016 P (Paul et al. 2017)	Single-center, Retrospective	Prediction of CR and OS to CRT for LA-EC	Multimodal	65	Manual Feature Engineering	GARF, Spearman’s rank analysis, RF classification, Genetic algorithm	5-fold cross-validation	GARF (RF)	NR	NR	Public	Open source
2	2019 Jin et al. (Jin et al., 2019)	Single-center, Retrospective	Prediction of response to CCRT for EC	Multimodal	94	IBEX, MATLAB	Pearson Correlation	Hold-out validation, 10-fold cross-validation, Stratified sampling	XGBoost	NR	NR	NR	NR
3	2020 Hu et al. (Hu et al., 2020)	Two-center, Retrospective	Prediction of pCR to NCRT for EC	Multimodal	231	PyRadiomics	Pearson Correlation, A wrapper method using recursive feature addition algorithm	External testing	SVM	NR	Y	NR	NR
4	2020 Rahman et al. (Rahman et al., 2020)	Multicenter, Retrospective	Prediction of early recurrence to NCRT/NCT for EAC/GEJ adenocarcinoma	Multimodal	812	NR	Regularization	Repeated 10-fold cross-validation, Internal–external validation, Bootstrap	Ensemble Combinations (ELR + RF + XGBoost)	Y	NR	NR	Open source
5	2020 Hu et al. (Hu et al., 2021)	Two-center, Retrospective	Prediction of pCR to NCRT for ESCC	Unimodal, Multimodal	231	Xception, VGG16, VGG19, ResNet50, InceptionV3, or InceptionResNetV2, PyRadiomics	Robustness, Univariate analysis, RFA	External testing	SVM	NR	70	NR	Open source
6	2021 Rishi et al. Rishi et al. (2021)	Single-center, Retrospective	Prediction of pCR to NCRT for ESCC	Unimodal, Multimodal	68	In-house data-characterization algorithm	Greedy conservative forward stepwise selection, Pearson correlation coefficient	LOOCV	Weka + LR	NR	NR	NR	NR
7	2021 Yoon et al. (Yoon et al., 2021)	Single-center, Retrospective	Prediction of excessive skeletal muscle loss to NACRT for ESCC	Multimodal	232	NR	NR	10-fold cross-validation, Bootstrap bias-corrected cross-validation	Ensemble Combinations (LR + SVM)	NR	NR	NR	NR
8	2022 Beukinga et al. (Beukinga et al., 2022)	Single-center, Retrospective	Prediction of non-response to NCRT for LA-EC	Multimodal	199	MATLAB 2018b	ICC, Pearson correlation, Principal component analysis, LASSO	5 repeated of 2-fold cross-validation, External validation	SVM	60	NR	Unavailable	Unavailable
9	2022 Huang et al. (Huang et al., 2022)	Single-center, Database, Retrospective	Prediction of interval from diagnosis to death to surgery, adjuvant RT/CT for ESCC	Unimodal	9069	NR	NR	Hold-out validation, External validation	DeepSurv (deep learning [DL] model)	383	NR	Available on request	Available on request
10	2022 Kawahara et al. (Kawahara et al., 2022)	Single-center, Retrospective	Prediction of pCR rate to NCRT for ESCC	Unimodal	98	NR	NR	Hold-out validation, 5-fold cross-validation	CNN	NR	NR	NR	NR
11	2022 Sasagawa et al. (Sasagawa et al., 2022)	Single-center, Retrospective	Prediction of OS and DFS to Platinum-based NCT for ESCC	Multimodal	121	NR	DT, RF classification	Cross-validation, Bootstrap, External validation	RF	20	NR	Public	Open source
12	2022 Yue et al. (Yue et al., 2022)	Two-center, Retrospective (Dataset A, B, C and D, Four datasets from Two centers in different periods)	Prediction of pCR to NCRT for ESCC	Unimodal	275	A medical imaging platform (www.miacsu.group)	RF classification	5-fold cross-validation, External validation	MLDRL (DL model)	Y	Y	NR	Open source
13	2023 Cheng et al. (Cheng et al., 2023)	Single-center, Retrospective	Prediction of response to CCRT for ESCC	Multimodal	105	PyRadiomics	Statistical differential analysis, LASSO, Mutual information (MI), Bootstrapping	Hold-out validation, 10-fold cross-validation	RF	NR	NR	Public	Open source
14	2023 Huang et al. (Huang et al., 2023)	Two-center, Retrospective	Prediction of ORR and PFS to treatment for inoperable elderly ESCC	Multimodal	189	PyRadiomics	ICC, mRMR	External validation	CTDEPN (Ensemble model)	20	NR	Available on request	NR
15	2023 Li et al. (Li et al., 2023a)	Single-center, Retrospective	Prediction of efficacy to neoadjuvant IC and post-IC for ESCC	Unimodal	95	PyRadiomics	Mann-Whitney U test, LASSO, ICC	5-fold cross-validation	RF	NR	NR	Available on request	Available on request
16	2023 Li et al. (Li et al., 2023b)	Multicenter, Retrospective	Prediction of pCR to NCRT for LA-ESCC	Unimodal, Multimodal	194	Pyradiomics	LASSO	5-fold cross-validation, External validation	SVM	Cohort 1: 51, Cohort 2: 27	NR	NR	NR
17	2023 Oda et al. (Oda et al., 2023)	Single-center, Retrospective	Prediction of pathological response to NAC for LA-EC	Unimodal	145	Radcloud	The variance threshold (variance threshold = 0.8), SelectKBest, LASSO	Hold-out validation, 5-fold cross-validation	LR	NR	NR	NR	NR
18	2023 Wang et al. (Wang et al., 2023)	Single-center, Retrospective	Prediction of pCR to NCRT for ESCC	Unimodal, Multimodal	112	PyRadiomics	RFE	LOOCV	LR	NR	NR	NR	NR
19	2023 Yap et al. (Yap et al., 2023)	Single-center, Retrospective	Prediction of responses to NCRT for ESCC	Multimodal	80	HRNetV2p	NR	5-fold cross-validation	HRNetV2p (CNN)	NR	NR	Available on request	NR
20	2024 Jung et al. (Jung et al., 2024)	Two-center, Retrospective	Prediction of LN status and tumor response to neoadjuvant FLOT for GEC	Multimodal	137	Xception (CNN)	NR	External validation	Xception (CNN)	59	NR	NR	NR
21	2024 Kasai et al. (Kasai et al., 2024)	Single-center, Retrospective	Prediction of response and prognosis to CRT for ESCC	Unimodal	44	MATLAB	C-statistics, Pearson’s correlation	Hold-out validation	RF	NR	NR	Available on request	Available on request
22	2023 Kawahara et al. (Kawahara et al., 2024)	Two-center, Retrospective	Prediction of local response to definitive RT for ESCC	Multimodal	142	PyRadiomics	Variance inflation factor (VIF), LASSO	5-fold cross-validation, External testing	NN	NR	26	NR	NR
23	2024 Li et al. (Li et al., 2024a)	Single-center, Retrospective	Prediction of clinical benefit to PD-1 inhibitors for ESCC	Unimodal	163	NR	NR	Hold-out validation	ViT-RNN network (RNN)	NR	NR	Available on request	Available on request
24	2024 Li et al. (Li et al., 2024b)	Two-center, Retrospective	Prediction of treatment outcome to NCRT for EC	Unimodal	231	NR	Student’s t-test, ICC, LASSO	10-fold cross-validation, External validation	XGBoost and RF	30	NR	NR	NR
25	2024 Lin et al. (Lin et al., 2024)	Two-center, Retrospective	Prediction of response to ICI and CT for ESCC	Unimodal, Multimodal	416	PyRadiomics	LASSO, Boratu, RFE	External validation	XGBoost	88	NR	NR	NR
26	2024 Liu et al. (Liu et al., 2024a)	Two-center, Retrospective	Prediction of pCR to NCRT for ESCC	Unimodal	155	PyRadiomics	Recursive feature elimination with 5-fold cross-validation (RFECV), ICC, Pearson correlation	5-fold cross-validation, External testing	RF	NR	73	Available on request	Open source
27	2024 Qi et al. (Qi et al., 2024)	Single-center, Retrospective design from prospective trials	Prediction of pCR to NCRT and anti-PD-1 inhibitors for ESCC	Unimodal, Multimodal	126	PyRadiomics	LASSO	K-fold cross-validation, 5-fold cross-validation	SVM	NR	NR	Available on request	Open source
28	2024 Su et al. (Su et al., 2024)	Single-center, Retrospective	Prediction of prognostic survival status to IT for EC	Multimodal	113	Filtering feature extraction	Filtering feature extraction, SelectKBest feature analysis	K-fold cross-validation, 10-fold cross-validation	Stacking ensemble model	NR	NR	NR	Open source
29	2024 Wang et al. (Wang et al., 2024)	Single-center, Retrospective	Prediction of pCR to NCRT and IT for ESCC	Unimodal, Multimodal	60	Medmind	RFE	5-fold cross-validation	XGBoost	NR	NR	Available on request	Available on request
30	2024 Zhang et al. (Zhang et al., 2024)	Two-center, Retrospective	Pridiction of pCR to NCRT for ESCC	Unimodal, Multimodal	190	AccuContour	ICC, mRMR, LASSO	Hold-out validation, 5-fold cross-validation, External validation	LR	33	NR	NR	NR

### 3.5 Quality assessment

Across a total of 56 items, TRIPOD + AI scores ranged from 23 to 46, with higher scores denoting greater transparency and reporting quality. When evaluated in relation to the aggregate number of TRIPOD + AI criteria implemented in each study, the median percentage of criteria fulfilled was 64.79% (IQR: 58.93%–71.89%, range: 47.92%–82.14%). Eleven articles completed more than 66.67% of the quality checklist (Supplementary Table A7), and almost all of research completed at least half of it (n = 29/30). The consistency of scores between reviewers was determined by Cohen’s Kappa, yielding satisfactory outcomes, indicating a more uniform evaluation between the two reviewers (mean: 0.9514, range: 0.7595-1.0000).

Current research on ML models for EC prediction showed strong adherence to foundational standards (Table 3), with 93.33% clearly defining predictors and 96.67% detailing data utilization, model construction, and internal validation, while most (96.67%) reported data collection context. However, key limitations persisted: transparency was lacking, with only 46.67% explaining prediction calculations, 40.00% sharing full datasets, and 23.33% providing model codes. Methodological justification was often insufficient, with only 26.67% justifying predictors selection, 16.67% addressing class imbalance, and 16.67% considering fairness (e.g., gender/racial bias). Multi-center studies rarely quantified data heterogeneity (35.71%), risking generalizability, and no post-publication updates (e.g., refinements or external validation) were reported as of October 2024 (Supplementary Tables A8-12).

**TABLE 3:** Compliance with reporting standards.

Bottom tertile (0–33.3, %)	Middle tertile (33.4–66.6, %)	Upper tertile (66.7–100, %)
Abstract-Registration (2f) [0]	Heterogeneity processing (12d) [35.71]	Evaluator qualification of subjective results (8b) [75]
Study size calculation (10) [0]	Blind assessment (8c) [37.93]	Prediction outcome (8a) [76.67]
Model updating (12f) [0]	Data sharing (18e) [40]	Model output (15) [76.67]
Protocol (18c) [0]	Heterogeneity in model performance (23b) [42.86]	Events number in modelling (21) [76.67]
Results updating (24) [0]	Abstract-Background (2a) [46.67]	Medical setting (6a) [80]
Users interaction (27b) [6.67]	Model predictions calculation (12g) [46.67]	Missing data (11) [80]
Registration (18d) [10]	Data flow/summary (20a) [55.17]	Abstract-Objectives (2b) [83.33]
Class imbalance (13) [16.67]	Evaluator qualification of subjective predictors (9c) [60.71]	Target population (3b) [83.33]
Discrimination (14) [16.67]	Multicenter differences (16) [62.5]	Treatment (6c) [83.33]
Code sharing (18f) [23.33]	Abstract-Methods (2c) [66.67]	Data pre-processing (7) [83.33]
Choice of predictors (9a) [26.67]	​	Title (1) [86.67]
Patient and public involvement (19) [30]	​	Objectives (4) [86.67]
Model details (22) [33.33]	​	Study date (5b) [86.67]
​	​	Eligibility criteria (6b) [86.67]
​	​	Model performance (23a) [86.67]
​	​	Unavailable data (27a) [86.67]
​	​	Funding (18a) [90]
​	​	Distribution of data characteristic (20c) [90]
​	​	Abstract-Results (2d) [93.33]
​	​	Defined predictors (9b) [93.33]
​	​	Predictors processing (12b) [93.33]
​	​	Conflicts of interest (18b) [93.33]
​	​	Future and promotion (27c) [93.33]
​	​	Data characteristic (20b) [96.55]
​	​	Abstract-Conclusion (2e) [96.67]
​	​	Data source (5a) [96.67]
​	​	Data utilization (12a) [96.67]
​	​	Modelling and internal validation (12c) [96.67]
​	​	Measurement for model performance (12e) [96.67]
​	​	Limitations (26) [96.67]
​	​	Medical context (3a) [100]
​	​	Ethics committee (17) [100]
​	​	Results interpretation (25) [100]

* The respective TRIPOD + AI, reference was shown in round brackets and the percentage of compliance is shown in square brackets.

## 4 Discussion

### 4.1 Summary of main findings

This systematic review analyzed 30 studies (2015-2024) on ML applications for esophageal cancer outcome prediction. Deep learning outperformed classical ML, with multimodal models surpassing single-modal approaches. Imaging data and clinical parameters emerged as the most prevalent data modalities, frequently integrated for multimodal predictive modeling. While supervised learning dominated, validation primarily employed k-fold cross-validation and external cohorts. TRIPOD + AI assessment (median 64.79% fulfillment rate) revealed gaps in computational transparency, predictor justification, and handling population heterogeneity, particularly in multicenter designs.

### 4.2 Challenges of ML models for medical prediction

Compared to other ML prediction domains, medical prediction models face unique challenges, characterized by three fundamental constraints: (1) inherent data complexities, including multi-modal integration requirements, vast lesion size variation, and limited data accessibility due to privacy regulations; (2) scarce of high-quality labeled data due to costly annotation; and (3) stringent regulatory requirements for clinical interpretability currently. Consequently, future medical prediction models requires focused attention on three strategic priorities: (1) the development of robust multi-modal fusion architectures capable of handling heterogeneous data types; (2) the implementation of scalable annotation frameworks that minimize labeling costs (e.g., AI-assisted pre-annotation tools), accelerate workflows (active learning with clinician-in-the-loop verification), and reduce annotator burden (automated quality control algorithms); and (3) the advancement of hybrid learning systems that embed medical domain knowledge and inherently interpretable structures balancing performance and clinical needs.

Medical ML faces challenges in acquiring large-scale labeled datasets due to costly manual annotation. While transfer learning from labeled source domains offers a solution, domain shifts and dataset bias limit effectiveness. Current approaches include fine-tuning (requiring target labels) and emerging paradigms like self-supervised/unsupervised learning to reduce annotation dependence. Unsupervised domain adaptation (UDA) shows promise for addressing cross-domain discrepancies in imaging protocols and populations, yet existing methods over-rely on statistical alignment strategies (e.g., adversarial training) while neglecting latent data knowledge in unlabeled data (Zhang et al., 2025). Future UDA architectures should dynamically adapt to clinical heterogeneity while preserving critical features.

### 4.3 Extended analysis

TabPFN (Tabular Prior-data Fitted Network), a foundational model tailored for small-to-medium tabular datasets, synergizes a Transformer encoder with in-context learning and structural causal modeling (Hollmann et al., 2025). It achieves state-of-the-art performance on datasets with ≤10,000 samples and 500 features, outperforming conventional methods (e.g., gradient-boosted trees) with only 2.8 s of inference time and 4 h of hyperparameter tuning—yielding orders-of-magnitude speedup. Its few-shot learning capability offers transformative potential for medical prediction tasks where data scarcity prevails.

Single-modality implementations primarily utilized clinical or imaging data independently, while multimodal systems’ performance gains likely originated from complementary cross-modal data integration, especially synergies between quantitative imaging features and clinical biomarkers.

Although current ML approaches show promise as tools to support clinical decisions in retrospective settings, there are still significant challenges to their routine clinical implementation. Future efforts should therefore focus on improving model interpretability using techniques such as SHAP values and heatmaps, in order to mitigate the “black box” concern and enhance clinician trust. In parallel, successful deployment requires seamless integration into existing clinical infrastructures, including electronic medical records and picture archiving and communication systems. It also requires the development of intuitive interfaces that translate model outputs into actionable insights while minimizing the cognitive burden on clinicians.

Future studies should prioritize multicenter collaborations with diverse population representation, prespecified subgroup analyses across demographic or histological strata, and federated learning frameworks to enhance global generalizability while addressing center-specific biases.

A substantial proportion of studies reviewed herein developed models based on radiomics (CT/PET/MR) data; however, radiomics data exhibit inherent limitations due to their dependence on variable image quality and feature extraction methodologies. Heterogeneity in scanner manufacturers, slice thickness, and reconstruction algorithms can induce intensity inhomogeneity, thereby compromising the stability of texture features (e.g., gray-level co-occurrence matrix, grey-level size-zone matrix), which may ultimately impair model performance and generalizability (Liu et al., 2024b).

Beyond technical limitations, deploying ML in clinical practice faces translational gaps, notably multi-center heterogeneity from divergent data collection and demographics (e.g., standard learning model, center-specific normalization), with few studies addressing performance disparities or fairness (e.g., gender/race biases). Transparent reporting of assessor demographics and bias-correction are needed. Missing data challenges are often inadequately handled via exclusion/imputation; a graded approach (e.g., removing variables with >50% missingness) may balance pragmatism and validity (Kawahara et al., 2024). Class imbalance could be typically addressed through data-level resampling techniques (e.g., minority class oversampling or majority class undersampling) and algorithm-level loss function weighting optimization.

External validation, the gold standard for assessing real-world model applicability, uses independent population data but may suffer from inter-institutional heterogeneity. Kawahara et al. (Kawahara et al., 2024) proposed a Hybrid Model strategy: pooling and randomly reallocating multi-institutional data (e.g., Institution 1 and 2) into training/testing sets to mitigate data barriers.

To address these challenges, standardized checklists (e.g., TRIPOD + AI or emerging frameworks like PROBAST-AI currently under development) should guide model development and validation by encompassing data quality, algorithmic fairness, and clinical applicability. A paradigm shift from static single-center models to dynamic systems accounting for clinical diversity and potential biases is essential, requiring sustained multi-institutional collaboration, transparent code/dataset sharing, and rigorous validation across heterogeneous populations—foundational steps for clinically robust ML tools in EC.

The future evolution of predictive models should focus on integrating dynamic imaging and biomarker data throughout the entire treatment course to construct longitudinal time-series prediction systems, moving beyond the constraints of unimodal radiomics and single-timepoint baseline data by deeply incorporating multi-omics data (genomics, immunomics, dosiomics) and developing efficient feature selection architectures supporting multimodal fusion. Looking ahead, oncological treatment prediction models will evolve from static single-center approaches towards interpretable, dynamic, multimodal, and clinically actionable frameworks, while systematically addressing critical challenges in data standardization, model transparency, and prospective validation to achieve translation from academic metric optimization into clinical decision support implementation.

### 4.4 Limitations

This study had some methodological limitations: First, English-only, recent-decade inclusion may introduce selection bias despite multi-database searches, excluding grey literature and trial registries. Second, heterogeneity in imaging modalities (CT/PET) and feature extraction methods, without standardized data transformation, biased cross-study comparisons. Third, approximately 50% of studies lacked algorithmic transparency (missing model construction details), preventing performance validation and limiting analysis to author-reported results. Fourth, the predominance of Asian populations, where ESCC is more common, may limit the generalizability of the included models to Western populations with higher rates of adenocarcinoma, and the lack of individual-level data precluded stratified analyses by race/ethnicity, sex, geographic region, and histological subtype, thereby constraining assessment of external validity. Finally, the heterogeneity in treatment regimens and model architectures limits the validity of direct quantitative comparisons across therapeutic contexts. Consequently, our heatmap visualization represents a descriptive synthesis of published data rather than a rigorous head-to-head evaluation. Future research should prioritize stratified development and external validation under standardized frameworks to better define model performance across specific clinical scenarios.

### 4.5 Conclusion

This review analyzed 30 studies (2015-2024, 14,342 EC patients) on ML-based pharmacological outcomes or responses prediction. DL outperformed classical and ensemble methods, with multimodal models surpassing single-modal approaches, while supervised learning and k-fold/external validation were predominant. TRIPOD + AI assessment (64.79% median compliance) identified critical gaps in code transparency, predictor justification, and the handling of population heterogeneity/class imbalance, especially in multicenter designs.

## Data Availability

The original contributions presented in the study are included in the article/Supplementary Material, further inquiries can be directed to the corresponding authors.

## Glossary

AdaB: Adaptive Boosting
ANN: Artificial Neural Network
AUC: Areas Under the Receiver Operating Characteristic Curve
CCRT: Concurrent Chemoradiotherapy
CI: Confidence Interval
CNN: Convolutional Neural Network
CPH: Cox Proportional Hazard
CR: Complete Response
CRT: Chemoradiotherapy
CT: Computed Tomography
CTDEPN: Combined Treatment Decision for Efficacy and Prognosis Nomogram
CTV: Clinical Target Volume
D-AAE: Disentangled-Multimodal adversarial autoencoder
DCR: Dose Contextual Representations
DFS: Disease-Free Survival
DL: Deep Learning
DT: Decision Tree
EAC: Esophageal Adenocarcinoma
EC: Esophageal Cancer
ECSS: Esophageal Cancer-Specific Survival
EFM: Early Fusion Method
ELR: Elastic Network Regression
EMR: Electronic Medical Record
ESCC: Esophageal Squamous Cell Carcinoma
ET: Extremely Randomized Trees
FPR: False Positive Rate
GBDT: Gradient Boosting Decision Trees
GEC: Gastroesophageal Cancer
GEJ: Gastroesophageal Junction
GTV: Gross Tumor Volume
HD-Net: HyperDense-Network
ICC: Interclass Correlation Coefficient
ICT: Immunochemotherapy
IT: Immunotherapy
ITH: Intratumor Heterogeneity
KNN: K-Nearest Neighbor
LA: Locally Advanced
LASSO: Least Absolute Shrinkage and Selection Operator
LFM: Late Fusion Method
LightGBM: Light Gradient Boosting
LN: Lymph Node
LOOCV: Leave-one-out Cross-validation
LR: Logistic Regression
MLDRL: Multi-loss Disentangled Representation Learning
MR: Magnetic Resonance
mRMR: Minimum Redundancy Maximum Relevance
NB: Naive Bayes
NCRT: Neoadjuvant Chemoradiotherapy
NCT: Neoadjuvant Chemotherapy
NN: Neural Network
nPCR: Nonpathological Complete Response
NPV: Negative Predictive Value
NR: Not Reported
ORR: Objective Response Rate
OS: Overall Survival
PCA: Principal Component Analysis
pCR: Pathological Complete Response
PD: Progressive Disease
PFS: Progression-Free Survival
PPV: Positive Predictive Value
PR: Partial Response
RF: Random Forest
RFA: Recursive Feature Addition
RFE: Recursive Feature Elimination
RFS: Recurrence-Free Survival
RNN: Recursive Neural Network
ROI: Region of Interest
RT: Radiotherapy
SD: Stable Disease
SEER: Surveillance, Epidemiology, and End Results
SHAP: SHapley Additive exPlanation
SIB: Simultaneous Integrated Boost
SMOTE: Synthetic Minority Over-sampling Technique
SVM: Support Vector Machine
TL: Transfer Learning
TPR: True Positive Rate
TRG: Tumor Regression Grade
TRIPOD: Transparent Reporting of a multivariable prediction model for Individual Prognosis Or Diagnosis
UDA: Unsupervised Domain Adaptation
WSI: Whole-Slide Image
XGBoost: Extreme Gradient Boosting
