# Supplementary material for: Machine learning applications in predicting pharmacological treatment outcomes and responses in esophageal cancer
Source: Front Pharmacol. 2026 Jun 17;17:1786601. doi: 10.3389/fphar.2026.1786601 (PMC13318985; doi:10.3389/fphar.2026.1786601)
Supplement: Supplementary file 1 [file Presentation1.pdf]

**Table A.1** Literature searching strategies and results

| Database | Searching Words | Results (2015-2024) |
|---|---|---|
| Pubmed | ((oesophagus OR gullet OR weasand OR esophageal OR gastro-esophageal OR gastroesophageal OR gastro-gesophageal OR upper gastrointestinal) AND (cancer OR tumor OR tumour OR oncology OR melanoma OR malignancy OR neoplasia OR adenocarcinoma OR squamous cell carcinoma)) AND (deep learning OR machine learning OR convolutional neural networks OR neural network OR algorithm OR artificial intelligence OR AI) AND (pharmacotherapy OR drug OR medication OR therapeutic OR chemotherapy OR chemical OR immunotherapy OR immunological OR immunization) AND (prognosis OR prediction) AND (respond OR response OR responsive OR outcome OR survive OR death OR adverse reaction OR ADR OR AE OR adverse effect OR toxicity) | 995 |
| Web of Science | (((((ALL=((oesophagus OR gullet OR weasand OR esophageal OR gastro-esophageal OR gastroesophageal OR gastro-gesophageal OR upper gastrointestinal))) AND ALL=((cancer OR tumor OR tumour OR oncology OR melanoma OR malignancy OR neoplasia OR adenocarcinoma OR squamous cell carcinoma))) AND ALL=((deep learning OR machine learning OR convolutional neural networks OR neural network OR algorithm OR artificial intelligence OR AI))) AND ALL=((pharmacotherapy OR drug OR medication OR therapeutic OR chemotherapy OR chemical OR immunotherapy OR immunological OR immunization))) AND ALL=((prognosis OR prediction))) AND ALL=((respond OR response OR responsive OR outcome OR survive OR death OR adverse reaction OR ADR OR AE OR adverse effect OR toxicity)) | 152 |
| IEEE Xplore | ("Full Text & Metadata":oesophagus OR "Full Text & Metadata":gullet OR "Full Text & Metadata":weasand OR "Full Text & Metadata":esophageal OR "Full Text & Metadata":gastro-esophageal OR "Full Text & Metadata":gastroesophageal OR "Full Text & Metadata":gastro-gesophageal OR "Full Text & Metadata":upper gastrointestinal) AND ("Full Text & Metadata":cancer OR "Full Text & Metadata":tumor OR "Full Text & Metadata":tumour OR "Full Text & Metadata":oncology OR "Full Text & Metadata":melanoma OR "Full Text & Metadata":malignancy OR "Full Text & Metadata":neoplasia OR "Full Text & Metadata":adenocarcinoma OR "Full Text & Metadata":squamous cell carcinoma) AND ("Full Text & Metadata":deep learning OR "Full Text & Metadata":machine learning OR "Full Text & Metadata":convolutional neural networks OR "Full Text & Metadata":neural network OR "Full Text & Metadata":algorithm OR "Full Text & Metadata":artificial intelligence OR "Full Text & Metadata":AI) AND ("Full Text & Metadata":pharmacotherapy OR "Full Text & Metadata":drug OR "Full Text & Metadata":medication OR "Full Text & Metadata":therapeutic OR "Full Text & Metadata":chemotherapy OR "Full Text & Metadata":chemical OR "Full Text & Metadata":immunotherapy OR "Full Text & Metadata":immunological OR "Full Text & Metadata":immunization) AND ("Full Text & Metadata":prognosis OR "Full Text & Metadata":prediction) AND ("Full Text & Metadata":respond OR "Full Text & Metadata":response OR "Full Text & Metadata":responsive OR "Full Text & Metadata":outcome OR "Full Text & Metadata":survive OR "Full Text & Metadata":death OR "Full Text & Metadata":adverse reaction | 414 |

| | | |
|---|---|---|
| | OR "Full Text & Metadata":ADR OR "Full Text & Metadata":AE OR "Full Text & Metadata":adverse effect OR "Full Text & Metadata":toxicity) | |
| ACM Digital Library | [[All: oesophagus] OR [All: gullet] OR [All: weasand] OR [All: esophageal] OR [All: gastro-esophageal] OR [All: gastroesophageal] OR [All: gastro-gesophageal] OR [All: upper gastrointestinal]] AND [[All: cancer] OR [All: tumor] OR [All: tumour] OR [All: oncology] OR [All: melanoma] OR [All: malignancy] OR [All: neoplasia] OR [All: adenocarcinoma] OR [All: squamous cell carcinoma]] AND [[All: deep learning] OR [All: machine learning] OR [All: convolutional neural networks] OR [All: neural network] OR [All: algorithm] OR [All: artificial intelligence] OR [All: ai]] AND [[All: pharmacotherapy] OR [All: drug] OR [All: medication] OR [All: therapeutic] OR [All: chemotherapy] OR [All: chemical] OR [All: immunotherapy] OR [All: immunological] OR [All: immunization]] AND [[All: prognosis] OR [All: prediction]] AND [[All: respond] OR [All: response] OR [All: responsive] OR [All: outcome] OR [All: survive] OR [All: death] OR [All: adverse reaction] OR [All: adr] OR [All: ae] OR [All: adverse effect] OR [All: toxicity]] | 532 |
| Cochrane library (Trials) | ((oesophagus OR gullet OR weasand OR esophageal OR gastro-esophageal OR gastroesophageal OR gastro-gesophageal OR upper gastrointestinal) AND (cancer OR tumor OR tumour OR oncology OR melanoma OR malignancy OR neoplasia OR adenocarcinoma OR squamous cell carcinoma)) AND (deep learning OR machine learning OR convolutional neural networks OR neural network OR algorithm OR artificial intelligence OR AI) AND (pharmacotherapy OR drug OR medication OR therapeutic OR chemotherapy OR chemical OR immunotherapy OR immunological OR immunization) AND (prognosis OR prediction) AND (respond OR response OR responsive OR outcome OR survive OR death OR adverse reaction OR ADR OR AE OR adverse effect OR toxicity) | 15 |
| Scopus | (TITLE-ABS-KEY(oesophagus OR gullet OR weasand OR esophageal OR gastro-esophageal OR gastroesophageal OR gastro-gesophageal OR upper-gastrointestinal) AND TITLE-ABS-KEY(cancer OR tumor OR tumour OR oncology OR melanoma OR malignancy OR neoplasia OR adenocarcinoma OR squamous-cell-carcinoma) AND ALL(deep-learning OR machine-learning OR convolutional-neural-networks OR neural-network OR algorithm OR artificial AND intelligence OR ai) AND ALL(pharmacotherapy OR drug OR medication OR therapeutic OR chemotherapy OR chemical OR immunotherapy OR immunological OR immunization) AND ALL(prognosis OR prediction) AND ALL(respond OR response OR responsive OR outcome OR survive OR death OR adverse-reaction OR adr OR ae OR adverse-effect OR toxicity)) | 604 |
| | Total | 2712 |

**Table A.2** PICOS framework for search criteria definition

| PICOS | Inclusion criteria | Exclusion criteria |
|---|---|---|
| **Population** | Human patients (adult) undergoing drug treatment for esophageal cancer. | Non-human participants (animals or modelling data generated algorithmically). |
| **Intervention** | Evaluating Artificial Intelligence Techniques/machine learning/deep learning in Patient Data; Prediction or prognosis of drug treatment outcomes or treatment responses directly related to esophageal cancer, treatment outcomes including survival time, death rates, treatment response including adverse events, drug adverse reaction, toxicity. | Not medication is used.<br>Application of AI to digital pathology and/or imaging, limited to a diagnostic purpose. |
| **Comparator** | Human clinicians or previously validated models. | NA |
| **Outcome** | Model performance/Effectiveness between AI prediction and control groups. Index: Accuracy, sensitivity, specificity, area under the curve (AUC) or receiver operating characteristic (ROC) curve of prediction. Any treatment-related outcomes: Treatment response, disease progression, survival time, death rate, toxicity. | Studies that did not show at least one of the following measures of classifier performance: accuracy, sensitivity, specificity, ROC or AUC. |
| **Study type** | Only primary studies comparing the performance of artificial intelligence versus clinicians/ previously validated models were included. Published with peer-reviewed in English before searching date. | Informal publication types (e.g., reviews, letters to the editor, editorials, conference abstracts, posters, commentaries, preprints, proposals). |

**Table A.3** Checklist for quality assessment modified from TRIPOD+AI

| Section/Topic | Item | Checklist item |
|---|---|---|
| **TITLE** | | |
| *Title* | 1 | Identify the study as developing or evaluating the performance of a multivariable prediction model, the target population, and the outcome to be predicted |
| **ABSTRACT** | | |
| *Abstract* | 2a | Background-Provide a brief explanation of the healthcare context and rationale for developing or evaluating the performance of all models |
| | 2b | Objectives-Specify the study objectives, including whether the study describes model development, evaluation, or both |
| | 2c | Methods-Describe the sources of data |
| | | Describe the eligibility criteria and setting where the data were collected |
| | | Specify the outcome to be predicted by the model, including time horizon of predictions in case of prognostic models |
| | | Specify the type of model, a summary of the model-building steps, and the method for internal validation |
| | | Specify the measures used to assess model performance (eg, discrimination, calibration, clinical utility) |
| | 2d | Results-Report the number of participants and outcome events |
| | | Summarize the predictors in the final model |
| | | Report model performance estimates (with confidence intervals) |
| | 2e | Conclusion-Give an overall interpretation of the main results |
| | 2f | Registration-Give the registration number and name of the registry or repository |
| **INTRODUCTION** | | |
| *Background* | 3a | Explain the healthcare context (including whether diagnostic or prognostic) and rationale for developing or evaluating the prediction model, including references to existing models |
| | 3b | Describe the target population and the intended purpose of the prediction model in the context of the care pathway, including its intended users (e.g., healthcare professionals, patients, public) |
| *Objectives* | 4 | Specify the study objectives, including whether the study describes the development or validation of a prediction model (or both) |
| **METHODS** | | |
| *Data* | 5a | Describe the sources of data separately for the development and evaluation datasets (e.g., randomized trial, cohort, routine care or registry data), the rationale for using these data, and representativeness of the data |
| | 5b | Specify the dates of the collected participant data, including start and end of participant accrual; and, if applicable, end of follow-up |
| *Participants* | 6a | Specify key elements of the study setting (e.g., primary care, secondary care, general population) including the number and location of centers |
| | 6b | Describe the eligibility criteria for study participants |
| | 6c | Give details of any treatments received, and how they were handled during model development or evaluation, if relevant |
| *Data preparation* | 7 | Describe any data pre-processing and quality checking, including whether this was similar across relevant sociodemographic groups |
| *Outcome* | 8a | Clearly define the outcome that is being predicted and the time horizon, including how and when assessed, the rationale for choosing this outcome, and whether the method of outcome assessment is consistent across sociodemographic groups |
| | 8b | If outcome assessment requires subjective interpretation, describe the qualifications and demographic characteristics of the outcome assessors |
| | 8c | Report any actions to blind assessment of the outcome to be predicted |
| *Predictors* | 9a | Describe the choice of initial predictors (e.g., literature, previous models, all available predictors) and any pre-selection of predictors before model building |

| | | |
|---|---|---|
| | 9b | Clearly define all predictors, including how and when they were measured (and any actions to blind assessment of predictors for the outcome and other predictors) |
| | 9c | If predictor measurement requires subjective interpretation, describe the qualifications and demographic characteristics of the predictor assessors |
| *Sample size* | 10 | Explain how the study size was arrived at (separately for development and evaluation), and justify that the study size was sufficient to answer the research question. Include details of any sample size calculation |
| *Missing data* | 11 | Describe how missing data were handled. Provide reasons for omitting any data |
| *Analytical methods* | 12a | Describe how the data were used (e.g., for development and evaluation of model performance) in the analysis, including whether the data were partitioned, considering any sample size requirements |
| | 12b | Depending on the type of model, describe how predictors were handled in the analyses (functional form, rescaling, transformation, or any standardization). |
| | 12c | Specify the type of model, rationale, all model-building steps, including any hyperparameter tuning, and method for internal validation |
| | 12d | Describe if and how any heterogeneity in estimates of model parameter values and model performance was handled and quantified across clusters (e.g., hospitals, countries). See TRIPOD-Cluster for additional considerations |
| | 12e | Specify all measures and plots used (and their rationale) to evaluate model performance (e.g., discrimination, calibration, clinical utility) and, if relevant, to compare multiple models |
| | 12f | Describe any model updating (e.g., recalibration) arising from the model evaluation, either overall or for particular sociodemographic groups or settings |
| | 12g | For model evaluation, describe how the model predictions were calculated (e.g., formula, code, object, application programming interface) |
| *Class imbalance* | 13 | If class imbalance methods were used, state why and how this was done, and any subsequent methods to recalibrate the model or the model predictions |
| *Fairness* | 14 | Describe any approaches that were used to address model fairness and their rationale |
| *Model output* | 15 | Specify the output of the prediction model (e.g., probabilities, classification). Provide details and rationale for any classification and how the thresholds were identified |
| *Training versus evaluation* | 16 | Identify any differences between the development and evaluation data in healthcare setting, eligibility criteria, outcome, and predictors |
| *Ethical approval* | 17 | Name the institutional research board or ethics committee that approved the study and describe the participant-informed consent or the ethics committee waiver of informed consent |
| **OPEN SCIENCE** | | |
| *Funding* | 18a | Give the source of funding and the role of the funders for the present study |
| *Conflicts of interest* | 18b | Declare any conflicts of interest and financial disclosures for all authors |
| *Protocol* | 18c | Indicate where the study protocol can be accessed or state that a protocol was not prepared |
| *Registration* | 18d | Provide registration information for the study, including register name and registration number, or state that the study was not registered |
| *Data sharing* | 18e | Provide details of the availability of the study data |
| *Code sharing* | 18f | Provide details of the availability of the analytical code |
| **PATIENT & PUBLIC INVOLVEMENT** | | |
| *Patient & Public Involvement* | 19 | Provide details of any patient and public involvement during the design, conduct, reporting, interpretation, or dissemination of the study or state no involvement. |
| **RESULTS** | | |
| *Participants* | 20a | Describe the flow of participants through the study, including the number of participants with and without the outcome and, if applicable, a summary of the follow-up time. A diagram may be helpful. |

| | | |
|---|---|---|
| | 20b | Report the characteristics overall and, where applicable, for each data source or setting, including the key dates, key predictors (including demographics), treatments received, sample size, number of outcome events, follow-up time, and amount of missing data. A table may be helpful. Report any differences across key demographic groups. |
| | 20c | For model evaluation, show a comparison with the development data of the distribution of important predictors (demographics, predictors, and outcome). |
| *Model development* | 21 | Specify the number of participants and outcome events in each analysis (e.g., for model development, hyperparameter tuning, model evaluation) |
| *Model specification* | 22 | Provide details of the full prediction model (e.g., formula, code, object, application programming interface) to allow predictions in new individuals and to enable third-party evaluation and implementation, including any restrictions to access or re-use (e.g., freely available, proprietary) |
| *Model performance* | 23a | Report model performance estimates with confidence intervals, including for any key subgroups (e.g., sociodemographic). Consider plots to aid presentation. |
| | 23b | If examined, report results of any heterogeneity in model performance across clusters. See TRIPOD Cluster for additional details. |
| *Model updating* | 24 | Report the results from any model updating, including the updated model and subsequent performance |
| **DISCUSSION** | | |
| *Interpretation* | 25 | Give an overall interpretation of the main results, including issues of fairness in the context of the objectives and previous studies |
| *Limitations* | 26 | Discuss any limitations of the study (such as a non-representative sample, sample size, overfitting, missing data) and their effects on any biases, statistical uncertainty, and generalizability |
| *Usability of the model in the context of current care* | 27a | Describe how poor quality or unavailable input data (e.g., predictor values) should be assessed and handled when implementing the prediction model |
| | 27b | Specify whether users will be required to interact in the handling of the input data or use of the model, and what level of expertise is required of users |
| | 27c | Discuss any next steps for future research, with a specific view to applicability and generalizability of the model |

**Table A.4** Data category and modality of data for prediction model training

| **Data Category** | **Number of Studies N=30** |
| --- | --- |
| **Image data** | n=23 (76.67%) |
| CT scans | n=20 |
| PET scans | n=6 |
| Endoscopic images | n=2 |
| MR radiomics | n=1 |
| **Clinical data** | n=18 (60.00%) |
| Clinical Information | n=18 |
| **Genomics data** | n=2 (6.67%) |
| DNA sequence | n=1 |
| RNA sequence | n=2 |
| **Lab data** | n=8 (26.67%) |
| Blood test | n=6 |
| WSI | n=2 |
| **Treatment data** | n=7 (23.33%) |
| Dositomics | n=3 |
| Immunomics | n=2 |

Abbreviations: CT, Computed Tomography; PET, Positron Emission Tomography; MR, Magnetic Resonance; WSI, Whole Slide Imaging.

**Table A.5** Data category and modality of data for multimodal training

| Multimodal Data Categories | Number of Studies |
|---|---|
| Different kinds of Image data | n=1 |
| Image data+Clinical data | n=7 |
| Image data+Genomics data | n=1 |
| Image data+Treatment data | n=3 |
| Clinical data+Treatment Data | n=1 |
| Clinical data+Lab data | n=2 |
| Image data+Clinical data+Lab data | n=4 |
| Clinical data+Genomics data+Treatment data | n=1 |
| Clinical data+Lab data+Treatment data | n=1 |

**Table A.6** Number of features extraction and selection

| Range of Feature Numbers | Initial Feature Extraction (n=30) | Final Feature Selection (n=30) |
|---|---|---|
| <100 | 4 (13.3%) | - |
| 101-200 | 2 (6.7%) | - |
| 201-500 | 2 (6.7%) | - |
| 501-1000 | 3 (10.0%) | - |
| 1001-2000 | 6 (20.0%) | - |
| >2000 | 5 (16.7%) | - |
| 0-10 | - | 12 (40.0%) |
| 11-20 | - | 8 (26.7%) |
| 21-40 | - | 0 (0%) |
| 41-60 | - | 2 (6.7%) |
| 61-80 | - | 0 (0%) |
| 81-100 | - | 1 (3.3%) |
| NR | 7 (23.3%) | 6 (20.0%) |
| Patches | 1 (3.3%) | 1 (3.3%) |

**Table A.7** Results of quality assessment and inter-rater agreement

| References | Score/Total (%) | Cohen 's Kappa |
|---|---|---|
| 2016 Paul et.al | 23/48 (47.92) | 0.9167 |
| 2019 Jin et.al | 32/53 (60.38) | 0.7595 |
| 2020 Hu et.al | 43/56 (76.79) | 1.0000 |
| 2020 Rahman et.al | 46/56 (82.14) | 1.0000 |
| 2020 Hu et.al | 42/56 (75.00) | 0.9512 |
| 2021 Rishi et.al | 34/53 (64.15) | 1.0000 |
| 2021 Yoon et.al | 36/51 (58.82) | 0.9592 |
| 2022 Beukinga et.al | 36/53 (67.92) | 0.9159 |
| 2022 Huang et.al | 30/56 (53.57) | 0.9278 |
| 2022 Kawahara et.al | 28/53 (52.83) | 1.0000 |
| 2022 Sasagawa et.al | 34/53 (64.15) | 0.9181 |
| 2022 Yue et.al | 33/56 (58.93) | 0.9634 |
| 2023 Cheng et.al | 41/56 (73.21) | 0.9554 |
| 2023 Huang et.al | 38/56 (67.86) | 1.0000 |
| 2023 Li et.al | 33/53 (62.26) | 1.0000 |
| 2023 Li et.al | 42/54 (77.78) | 0.9480 |
| 2023 Oda et.al | 36/53 (67.92) | 1.0000 |
| 2023 Wang et.al | 32/53 (60.38) | 1.0000 |
| 2023 Yap et.al | 30/53 (56.60) | 0.9618 |
| 2024 Jung et.al | 33/56 (58.93) | 0.9629 |
| 2024 Kasai et.al | 35/53 (66.04) | 0.9180 |
| 2023 Kawahara et.al | 35/56 (62.50) | 0.9615 |
| 2024 Li et.al | 33/53 (62.26) | 0.9594 |
| 2024 Li et.al | 34/56 (60.71) | 0.9629 |
| 2024 Lin et.al | 43/56 (76.79) | 1.0000 |
| 2024 Liu et.al | 44/56 (78.57) | 1.0000 |
| 2024 Qi et.al | 33/56 (58.93) | 0.9629 |
| 2024 Su et.al | 28/53 (52.83) | 0.8466 |
| 2024 Wang et.al | 35/56 (62.50) | 0.8868 |
| 2024 Zhang et.al | 42/56 (75.00) | 0.9048 |

**Table A.8** Reviewer TRIPOD+AI title, abstract and background checklist

| Section | Topic | Checklist item | Total | Inter-rater agreement |
|---|---|---|---|---|
| | | | N=30 | Cohen's Kappa |
| Title | Title | Identify the study as developing or evaluating the performance of a multivariable prediction model, the target population, and the outcome to be predicted | 26 (86.67%) | 0.8387 |
| Abstract | Background | Provide a brief explanation of the healthcare context and rationale for developing or evaluating the performance of all models | 14 (46.67%) | 0.8000 |
| | Objectives | Specify the study objectives, including whether the study describes model development, evaluation, or both | 25 (83.33%) | 0.8696 |
| | Methods | Describe the sources of data<br>Describe the eligibility criteria and setting where the data were collected<br>Specify the outcome to be predicted by the model, including time horizon of predictions in case of prognostic models<br>Specify the type of model, a summary of the model-building steps, and the method for internal validation<br>Specify the measures used to assess model performance (eg, discrimination, calibration, clinical utility) | 20 (66.67%) | 0.8507 |
| | Results | Report the number of participants and outcome events<br>Summarise the predictors in the final model<br>Report model performance estimates (with confidence intervals) | 28 (93.33%) | 1.0000 |
| | Conclusion | Give an overall interpretation of the main results | 29 (96.67%) | 1.0000 |
| | Registration | Give the registration number and name of the registry or repository | 0 (0%) | 1.0000 |
| Introduction | Background | Explain the healthcare context (including whether diagnostic or prognostic) and rationale for developing or evaluating the prediction model, including references to existing models | 30 (100%) | 1.0000 |
| | Background | Describe the target population and the intended purpose of the prediction model in the context of the care pathway, including its intended users (e.g., healthcare professionals, patients, public) | 25 (83.33%) | 0.8696 |
| | Objectives | Specify the study objectives, including whether the study describes the development or validation of a prediction model (or both) | 26 (86.67%) | 0.8387 |

The majority of studies identified the prediction model, objectives, target population and outcomes in the title, abstract and background. Approximately two-thirds studies provided an explainable method in abstracts (n=20/30, 66.67%). Less than half of the studies detailed the context of the study in their abstracts (n=14/30, 46.67%). No study demonstrated the registration information in the abstract.

**Table A.9** Reviewer TRIPOD+AI data collection and characteristics checklist

| Section | Topic | Checklist item | Total | Inter-rater agreement |
|---|---|---|---|---|
| | | | N=30-NA | Cohen's Kappa |
| Methods | Data | Describe the sources of data separately for the development and evaluation datasets (e.g., randomised trial, cohort, routine care or registry data), the rationale for using these data, and representativeness of the data | 29/30 (96.67%) | 1.0000 |
| | Data | Specify the dates of the collected participant data, including start and end of participant accrual; and, if applicable, end of follow-up | 26/30 (86.67%) | 1.0000 |
| | Participants | Specify key elements of the study setting (e.g., primary care, secondary care, general population) including the number and location of centres | 24/30 (80.00%) | 1.0000 |
| | Participants | Describe the eligibility criteria for study participants | 26/30 (86.67%) | 0.8696 |
| | Participants | Give details of any treatments received, and how they were handled during model development or evaluation, if relevant | 25/30 (83.33%) | 1.0000 |
| | Data preparation | Describe any data pre-processing and quality checking, including whether this was similar across relevant sociodemographic groups | 25/30 (83.33%) | 0.5946 |
| | Outcome | Clearly define the outcome that is being predicted and the time horizon, including how and when assessed, the rationale for choosing this outcome, and whether the method of outcome assessment is consistent across sociodemographic groups | 23/30 (76.67%) | 0.9112 |
| | Outcome | If outcome assessment requires subjective interpretation, describe the qualifications and demographic characteristics of the outcome assessors | 21/28 (75.00%) | 0.9000 |
| | Outcome | Report any actions to blind assessment of the outcome to be predicted | 11/29 (37.93%) | 1.0000 |
| | Predictors | If predictor measurement requires subjective interpretation, describe the qualifications and demographic characteristics of the predictor assessors | 17/28 (60.71%) | 1.0000 |
| | Sample size | Explain how the study size was arrived at (separately for development and evaluation), and justify that the study size was sufficient to answer the research question. Include details of any sample size calculation | 0/30 (0%) | |
| | Missing data | Describe how missing data were handled. Provide reasons for omitting any data | 24/30 (80.00%) | 1.0000 |
| | Unavailable data | Describe how poor quality or unavailable input data (e.g., predictor values) should be assessed and handled when implementing the prediction model | 26/30 (86.67%) | 0.8696 |
| | Training versus evaluation | Identify any differences between the development and evaluation data in healthcare setting, eligibility criteria, outcome, and predictors | 10/16 (62.5%) | 1.0000 |

The preponderance of research had focused on delineating the source and temporal context of clinical data collection (n=29/30, 96.67%; n=26/30, 86.67%), in addition to the eligibility criteria for study participants (n=26/30, 86.67%). About one-third of the studies reported the measures to blind assessment of the outcomes (n=11/29, 37.93%). There was moderate disagreement among reviewers on data preparation issues ($\kappa$=0.5946). It was noteworthy that no study provided a comprehensive explanation of how to ascertain and calculate the study size. It would appear that there was inadequate disclosure of information for the identity qualifications of assessors regarding subjective predictors and treatment outcomes (e.g., medical imaging information) (n=17/28, 60.71%; n=21/28, 75.00%). In addressing with missing and unavailable data, most of the articles provided specific explanations and descriptions of appropriate methodologies (n=24/30, 80.00%; n=26/30, 86.67%). Most studies directly excluded unavailable and poor-quality data, and a few used corresponding medians or means padding the vacancy,

especially for small-size studies. With regard to the research conducted across multiple centers, more than half of the studies identified disparities or examined the consistency of data or the characteristics of participants across centers (n=10/16, 62.5%).

**Table A.10** Reviewer TRIPOD+AI model construction and algorithm transparency checklist

| Section | Topic | Checklist item | Total | Inter-rater agreement |
|---|---|---|---|---|
| | | | N=30-NA | Cohen's Kappa |
| Methods | Predictors | Describe the choice of initial predictors (e.g., literature, previous models, all available predictors) and any pre-selection of predictors before model building | 8/30 (26.67%) | 1.0000 |
| | Predictors | Clearly define all predictors, including how and when they were measured (and any actions to blind assessment of predictors for the outcome and other predictors) | 28/30 (93.33%) | 1.0000 |
| | Analytical methods | Describe how the data were used (e.g., for development and evaluation of model performance) in the analysis, including whether the data were partitioned, considering any sample size requirements | 29/30 (96.67%) | 1.0000 |
| | Analytical methods | Depending on the type of model, describe how predictors were handled in the analyses (functional form, rescaling, transformation, or any standardization). | 28/30 (93.33%) | 1.0000 |
| | Analytical methods | Specify the type of model, rationale, all model-building steps, including any hyperparameter tuning, and method for internal validation | 29/30 (96.67%) | 1.0000 |
| | Analytical methods | Describe if and how any heterogeneity in estimates of model parameter values and model performance was handled and quantified across clusters (e.g., hospitals, countries). | 5/14 (35.71%) | 0.8511 |
| | Analytical methods | Specify all measures and plots used (and their rationale) to evaluate model performance (e.g., discrimination, calibration, clinical utility) and, if relevant, to compare multiple models | 29/30 (96.67%) | 1.0000 |
| | Analytical methods | Describe any model updating (e.g., recalibration) arising from the model evaluation, either overall or for particular sociodemographic groups or settings | 0/30 (0%) | 1.0000 |
| | Analytical methods | For model evaluation, describe how the model predictions were calculated (e.g., formula, code, object, application programming interface) | 14/30 (46.67%) | 1.0000 |
| | Class imbalance | If class imbalance methods were used, state why and how this was done, and any subsequent methods to recalibrate the model or the model predictions | 5/30 (16.67%) | 1.0000 |
| | Fairness | Describe any approaches that were used to address model fairness and their rationale | 5/30 (16.67%) | 1.0000 |
| | Model output | Specify the output of the prediction model (e.g., probabilities, classification). Provide details and rationale for any classification and how the thresholds were identified | 23/30 (76.67%) | 0.7917 |
| Open Science | Data sharing | Provide details of the availability of the study data | 12/30 (40.00%) | 0.9315 |
| | Code sharing | Provide details of the availability of the analytical code | 7/30 (23.33%) | 1.0000 |
| Results | Model development | Specify the number of participants and outcome events in each analysis (e.g., for model development, hyperparameter tuning, model evaluation) | 23/30 (76.67%) | 1.0000 |
| | Model specification | Provide details of the full prediction model (e.g., formula, code, object, application programming interface) to allow predictions in new individuals and to enable third-party evaluation and implementation, including any restrictions to access or re-use (e.g., freely available, proprietary) | 10/30 (33.33%) | 1.0000 |
| | Model performance | Report model performance estimates with confidence intervals, including for any key subgroups (e.g., sociodemographic). Consider plots to aid presentation. | 26/30 (86.67%) | 0.7143 |
| | Model performance | If examined, report results of any heterogeneity in model performance across clusters. | 6/14 (42.86%) | 0.8511 |
| | Model updating | Report the results from any model updating, including the updated model and subsequent performance | 0/30 (0%) | 1.0000 |

| | | | | |
|---|---|---|---|---|
| Discussion | Usability of the model in the context of current care | Specify whether users will be required to interact in the handling of the input data or use of the model, and what level of expertise is required of users | 2/30 (6.67%) | 1.0000 |

As demonstrated in **Table A.10**, contrary to the majority of extant studies, which had published definitions and measurements of predictors (n=28/30, 93.33%), fewer studies adequately explained in detail the reasons and evidence for the selection of predictors (n=8/30, 26.67%). The majority of studies described the utilization of data (n=29/30, 96.67%), the processing of predictors (n=28/30, 93.33%), the construction of a model and the internal validation (n=29/30, 96.67%), and evaluation of model performance (n=29/30, 96.67%). At the time of assessment (December 2024), there were no studies publishing model-related updates and reports. Researchers indicated that the majority of studies had focused on the output of the prediction model (n=23/30, 76.67%), yet only a limited number of studies have provided the details and underlying rationale for the threshold. The majority of studies had reported model performance estimates alongside confidence intervals (CI), or relevant key subgroups. For algorithm transparency, less than half of studies minutely described how the model predictions were calculated (e.g., formula, code) (n=14/30, 46.67%). A paucity of studies had published detailed or fully exposed data particulars (n=12/30, 40.00%) and the model codes (n=7/30, 23.33%) for their study.

For data collected across clusters (e.g. hospitals, countries) from multiple centers, only five studies stated how heterogeneity was handled and quantified (n=5/14, 35.71%). Identifying and dealing with class imbalance data is necessary for machine learning in general, and classification models in particular, to improve model confidence. It is noted that only a small number of studies explicitly justify and correct for class imbalance (n=5/30, 16.67%). In the literature, there is a lack of attention to the description of methodologies employed to address issues of fairness (e.g., gender, race) in models and rationales (n=5/30, 16.67%). Only two studies mentioned the interaction in the handling of the input data or utilization of the model for potential users.

In this part of the examination of the checklist, there was a more uniform consistency of opinion among the researchers (range of κ: 0.7143-1.000, IQR: 0.9829-1.0000).

**Table A.11** Reviewer TRIPOD+AI results interpretation and discussion checklist

| Section | Topic | Checklist item | Total | Inter-rater agreement |
|---|---|---|---|---|
| | | | N=30-NA | Cohen's Kappa |
| Results | Participants | Describe the flow of participants through the study, including the number of participants with and without the outcome and, if applicable, a summary of the follow-up time. A diagram may be helpful. | 16/29 (55.17%) | 0.9298 |
| | Participants | Report the characteristics overall and, where applicable, for each data source or setting, including the key dates, key predictors (including demographics), treatments received, sample size, number of outcome events, follow-up time, and amount of missing data. A table may be helpful. Report any differences across key demographic groups. | 28/29 (96.55%) | 0.6506 |
| | Participants | For model evaluation, show a comparison with the development data of the distribution of important predictors (demographics, predictors, and outcome). | 27/30 (90.00%) | 0.4643 |
| Discussion | Interpretation | Give an overall interpretation of the main results, including issues of fairness in the context of the objectives and previous studies | 30/30 (100%) | 1.0000 |
| | Limitations | Discuss any limitations of the study (such as a non-representative sample, sample size, overfitting, missing data) and their effects on any biases, statistical uncertainty, and generalizability | 29/30 (96.67%) | 1.0000 |
| | Usability of the model in the context of current care | Discuss any next steps for future research, with a specific view to applicability and generalizability of the model | 28/30 (93.33%) | 0.3662 |

More than 90% of the studies described and quantified the characteristics and distribution of patients, but only about half of the studies described the flow of participants through the trials in more details, especially using flowcharts (n=16/29, 55.17%). Almost all studies included an overall summary and discussion of findings and limitations. Future studies and expansion were also discussed (n=28/30, 93.33%).

**Table A.12** Reviewer TRIPOD+AI disclosure of information checklist

| Section | Topic | Checklist item | Total | Inter-rater agreement |
|---|---|---|---|---|
| | | | **N=30-NA** | **Cohen's Kappa** |
| Open Science | Ethical approval | Name the institutional research board or ethics committee that approved the study and describe the participant-informed consent or the ethics committee waiver of informed consent | 30/30 (100%) | 1.0000 |
| | Funding | Give the source of funding and the role of the funders for the present study | 27/30 (90.00%) | 1.0000 |
| | Conflicts of interest | Declare any conflicts of interest and financial disclosures for all authors | 28/30 (93.33%) | 1.0000 |
| | Protocol | Indicate where the study protocol can be accessed or state that a protocol was not prepared | 0/30 (0%) | |
| | Registration | Provide registration information for the study, including register name and registration number, or state that the study was not registered | 3/30 (10.00%) | 0.7826 |
| Patient & Public Involvement | Patient & Public Involvement | Provide details of any patient and public involvement during the design, conduct, reporting, interpretation, or dissemination of the study or state no involvement. | 9/30 (30.00%) | 0.9180 |

All authors had ethics committee review and informed consent, or retrospective studies did not require patient consent (1 used data from a published study which had also passed ethics review). The most published studies presented information on funding and conflicts. Publication of registration information should be encouraged as few studies provided protocol (n=0/30) and registration details (n=3/30, 10.00%). There was insufficient reporting of public involvement (n=9/30, 30.00%), and most disclosure of involvement focused on professional industry experts and companies involved in modelling process, rather than patients.

**Fig A.13** Deek' s Funnel Plot asymmetry test of AUC in included studies

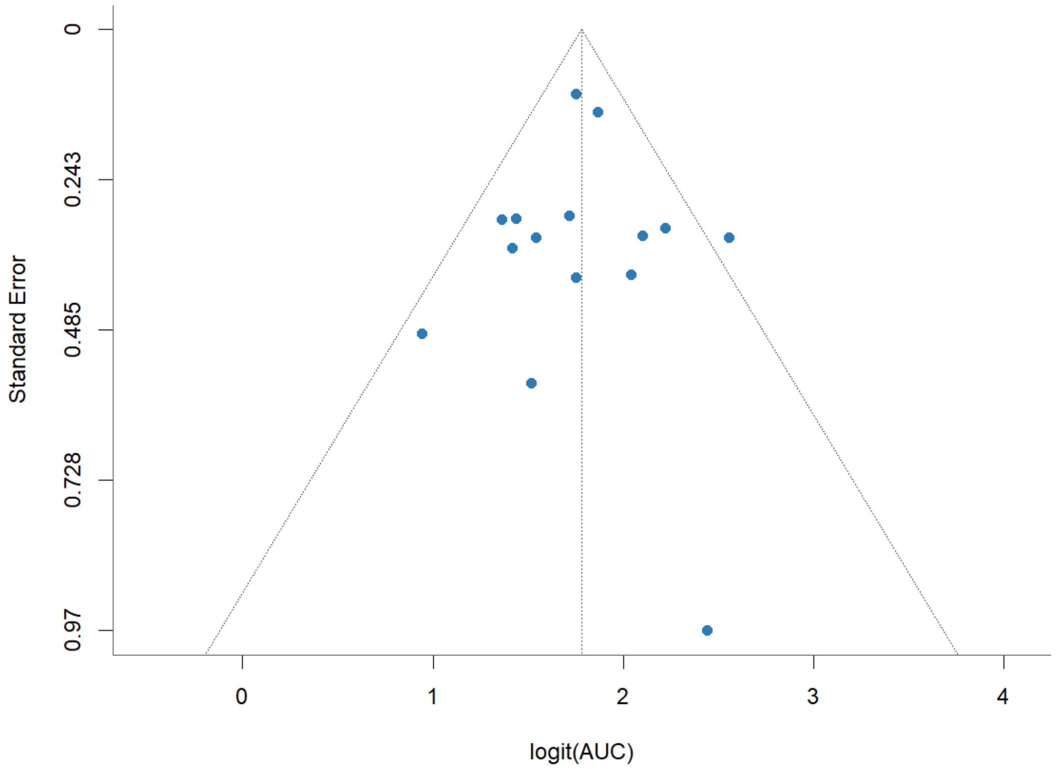


A detailed assessment of publication bias was conducted by plotting Deek's Funnel Plot asymmetry test. Although there was a certain degree of dispersion, it showed no obvious asymmetry and no situation where a large number of studies were missing on one side, indicating that in the set of studies included in this review, the influence of publication bias was likely to be small.
